# Traumatic Submacular Hemorrhage with Macular Hole Repaired by Pneumatic Displacement and Intravitreal t-PA Injection

**DOI:** 10.1155/2017/9821416

**Published:** 2017-06-11

**Authors:** Amy A. Mehta, Jay Berdia, Lilly H. Wagner, Irene Choi, Brandon B. Johnson

**Affiliations:** Bronx-Lebanon Hospital Center, Bronx, NY, USA

## Abstract

This paper presents a case demonstrating repair of traumatic macular hole and submacular hemorrhage with intravitreal gas tamponade and t-PA in an office setting.

## 1. Introduction

Sequelae of traumatic eye injury, including submacular hemorrhage (SMH) and macular hole (MH), are well described and can be visually devastating [1, 2]. Various treatment options for SMH have been studied including intravitreal injection of antivascular endothelial growth factor (VEGF), photodynamic therapy, pneumatic displacement, or pars plana vitrectomy (PPV) with or without adjuvant intravitreal tissue plasminogen activator (t-PA) [3]. Traumatic MH repair frequently involves PPV with or without peeling of the internal limiting membrane and gas tamponade. Historically, intravitreal t-PA has been reserved for the treatment of conditions that induce choroidal neovascularization (CNV) such as exudative age related macular degeneration (AMD); however its utility in the management of traumatic SMH has been increasingly documented [2, 4, 5]. This case demonstrates combined displacement of a traumatic SMH and repair of a MH with intravitreal perfluoropropane (C3F8) and t-PA.

## 2. Case Report

**Figure 1:**
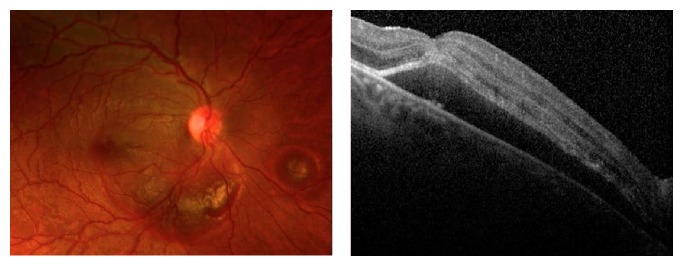
Fundus photograph and Spectral-Domain Optical Coherence Tomography (SD-OCT) of the right eye two days after presentation demonstrating submacular hemorrhage (SMH) involving the fovea.

A 27-year-old male with no past medical history presented to the Emergency Department after sustaining blunt trauma to his right eye secondary to an airbag injury. Visual acuity measured 20/400 in the affected eye. Funduscopic examination demonstrated an arcuate choroidal rupture inferior to the optic nerve and subretinal hemorrhage extending from the optic nerve into the fovea (Figure 1).

**Figure 2:**
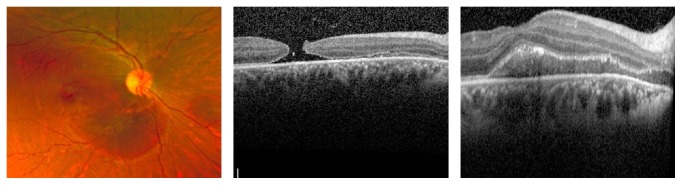
Fundus photograph and Spectral-Domain Optical Coherence Tomography (SD-OCT) of the right eye one week after presentation demonstrating macular hole (MH) and inferior submacular hemorrhage (SMH).

One week later, the patient's visual acuity in the affected eye had declined to counting fingers. Funduscopic examination revealed a newly developed, full thickness macular hole with persistent inferior SMH (Figure 2). The patient underwent office-based intravitreal injection of 0.05 mL of t-PA (50 *µ*g/0.05 mL) and 0.25 cc of C3F8 gas.

Throughout the postoperative course the patient required both anterior chamber and vitreous tap (postinjection days 2 and 4, resp.) to manage elevated intraocular pressure secondary to partial migration of the gas bubble into the anterior chamber, presumably due to zonular dehiscence.

**Figure 3:**
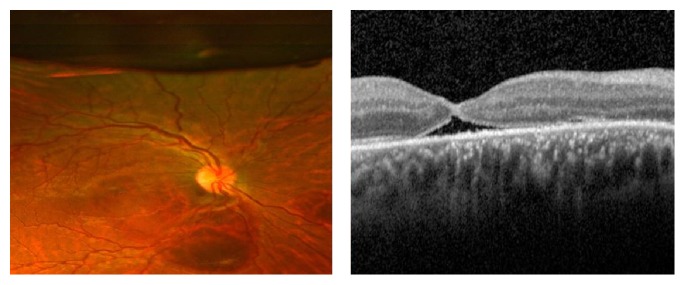
Fundus photograph and Spectral-Domain Optical Coherence Tomography (SD-OCT) of the right eye three weeks after intravitreal injection of C3F8 gas and t-PA demonstrating closure of macular hole (MH) and inferior displacement of submacular hemorrhage (SMH).

**Figure 4:**
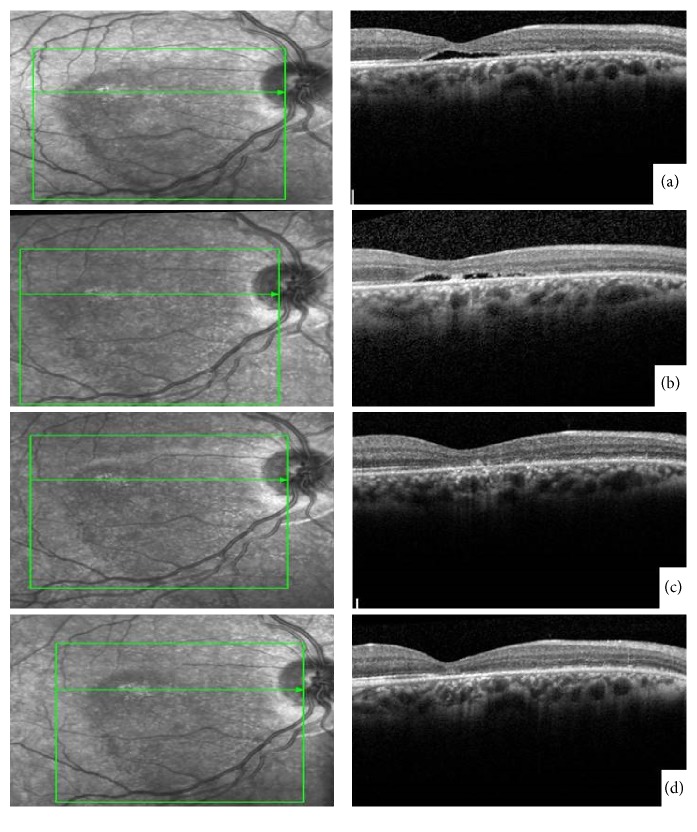
Spectral-Domain Optical Coherence Tomography (SD-OCT) progression demonstrating resolution of the macular hole (MH) and regeneration of outer retinal layers one (a), two (b), three (c), and five months' (d) status after pneumatic displacement and t-PA intravitreal injection.

Subsequent clinic visits showed successful displacement of subretinal blood out of the macula and resolution of the macular hole (Figures 3 and 4). Four months after intervention, the patient's best corrected visual acuity had improved to 20/70.

## 3. Discussion

Complications of ocular trauma include subretinal hemorrhage, macular hole, CNV, retinal detachment, and other potentially sight-threatening conditions. SMH is a potential visually devastating condition, most commonly associated with CNV due to AMD [1, 5, 6]. Subretinal blood and blood products damage retinal tissues through the toxic effects of iron, hemosiderin, and fibrin on the overlying photoreceptors. In addition, clot retraction can sheer and damage the photoreceptors while the physical separation of the photoreceptors from the RPE can lead to profound retinal dysfunction [1, 2, 5]. Management of SMH has been described using multiple approaches alone or in combination with varying success including observation, PPV, pneumatic displacement, t-PA, and anti-VEGF [4–6].

While spontaneous closure of traumatic macular holes has been described, successful closure frequently requires intervention [7]. Surgical options for traumatic macular hole repair include PPV with gas or silicon oil tamponade as well as adjuvant therapies such as plasmin, platelets, autologous serum, and transforming growth factor. In our case of traumatic macular hole, the presence of concurrent, slowly resolving SMH was considered a poor prognostic factor for final visual outcome.

The goal of pneumatic displacement in cases of SMH is to physically remove subretinal hemorrhage from the fovea using a combination of expansile gas and posturing. Under topical anesthesia and sterile conditions, intravitreal injection of either short-acting sulfur hexafluoride (SF6) or longer-acting perfluoropropane (C3F8) is performed and followed by face-down head positioning for one to three days. Complications of intravitreal injection include endophthalmitis, vitreous hemorrhage, and retinal detachment. Case series have shown that the ideal candidates for pneumatic displacement are those with thick SMH less than three weeks old involving or inferior to the fovea [1, 2]. There is evidence suggesting the addition of 0.1 to 0.2 mL of intravitreal t-PA (either 25 *μ*g or 50 *μ*g/mL) can augment clot displacement presumably through clot liquefaction [5, 8]. Potential adverse events related to t-PA injection include retinal toxicity and exudative retinal detachment. The visual outcomes of patients with traumatic SMH are variable despite treatment options and largely dependent on the degree of traumatic injury [5, 9].

The treatment of combined SMH and MH secondary to trauma is challenging as the rarity of the condition precludes an abundance of level one data to guide management. Reports suggest PPV or pneumatic displacement with or without adjunctive t-PA may improve anatomic and visual outcome; however possible side effects and complications of these interventions including cataract, retinal toxicity, and endophthalmitis must be considered [1, 10]. Other sequelae of trauma such as zonular dialysis, microhyphema, and elevated intraocular pressure must be identified as they may complicate treatment. To our knowledge this is the first report of traumatic submacular hemorrhage with full thickness macular hole treated successfully with pneumatic displacement and intravitreal injection of t-PA.
